# Rentrop collateral grade predicts myocardial viability in chronic total occlusion on cardiac magnetic resonance

**DOI:** 10.1136/openhrt-2025-003930

**Published:** 2026-05-27

**Authors:** Zia Mehmood, Preethi Suresh, Rui Li, Hosamadin Assadi, Alexander Gall, Bahman Kasmai, Kurian Thampi, Chris Sawh, Rob J van der Geest, Alisdair Ryding, Gareth Matthews, Pankaj Garg

**Affiliations:** 1 Cardiology, Norfolk and Norwich University Hospitals NHS Foundation Trust, Norwich, UK; 2 Department of Cardiovascular and Metabolic Health, University of East Anglia, Norwich, UK; 3 Norfolk and Norwich University Hospitals NHS Foundation Trust, Norwich, UK; 4 Leiden University Medical Center (LUMC), Leiden, Netherlands; 5 University of East Anglia, Norwich, UK

**Keywords:** Magnetic Resonance Imaging, Coronary Artery Disease, Myocardial Infarction, Percutaneous Coronary Intervention

## Abstract

**Background:**

Chronic total occlusion (CTO) often triggers collateral coronary development, yet the relationship between angiographically graded collaterals and myocardial viability remains incompletely understood. We evaluated whether Rentrop collateral grading predicts tissue-level viability using contemporary quantitative cardiac magnetic resonance (CMR).

**Methods:**

We enrolled 56 patients with angiographically confirmed CTO who underwent CMR. Collateral flow was graded using the Rentrop classification. Viable myocardium was defined as ≤50% transmural scar on late gadolinium enhancement. Quantitative myocardial blood flow (MBF) and scar burden were assessed within CTO territories.

**Results:**

Higher Rentrop grades were linked to greater myocardial viability (Rentrop 0: 44.4% viable; Rentrop 2: 88.9% viable; p=0.043). CTO-territory stress MBF differed across Rentrop grades (p=0.04). In multivariable analysis, Rentrop grade emerged as the only independent predictor of viability (OR 2.52, 95% CI 1.18 to 5.42, p=0.01). Receiver operating characteristic analysis showed an area under the curve of 0.71, identifying Rentrop >1 as the optimal cut-off for predicting viability (sensitivity 66%, specificity 73%, p=0.01). In addition, there was a significant association between Canadian Cardiovascular Society anginal severity and collateral development (χ²=8.548, p=0.035).

**Conclusions:**

Angiographic grading of collaterals using the Rentrop score independently predicts myocardial viability in patients with CTO. These findings support integrating collateral assessment into risk stratification and revascularisation planning, particularly when advanced imaging is limited.

**WHAT IS ALREADY KNOWN ON THIS TOPIC**

- Chronic total occlusion (CTO) often induces collateral coronary development, which may preserve myocardial viability. However, the link between angiographic collateral grading (eg, Rentrop score) and myocardial viability as assessed by advanced imaging like cardiovascular magnetic resonance is poorly understood. This uncertainty complicates revascularisation decisions, especially when advanced imaging is unavailable.

**WHAT THIS STUDY ADDS**

- Predictive accuracy: Rentrop collateral grading is an independent predictor of myocardial viability with higher Rentrop grades corresponding to greater myocardial preservation (OR 2.52, 95% CI 1.18 to 5.42, p=0.01). Threshold Insight: Rentrop score >1 is the optimal cut-off for identifying viable myocardium (sensitivity 66%, specificity 73%, area under the curve 0.71, p=0.01), offering a practical and clinically useful tool for decision-making in percutaneous coronary intervention (PCI), particularly when symptoms and ischaemia are in agreement.

**HOW THIS STUDY MIGHT AFFECT RESEARCH, PRACTICE OR POLICY**

- Clinical adoption: Emphasises the importance of incorporating Rentrop grading into CTO risk assessment and revascularisation strategies, enhancing patient selection for PCI. Guideline evolution: Advocates for the inclusion of collateral grading in CTO management protocols and calls for further research to standardise phenotyping and patient selection.

## Introduction

 Chronic total occlusion (CTO) is a frequent finding at diagnostic angiography, seen in about one in five patients with established coronary artery disease.^1^ It reflects healed plaque rupture with thrombus organisation and adverse remodelling that abolishes antegrade flow, most often in the right coronary artery.^1 2^ The main clinical question in CTO is which patients should undergo revascularisation. Percutaneous coronary intervention (PCI) for CTO is feasible in many centres, but procedures remain long and resource-intensive. Randomised trials provide a consistent signal of better disease-specific health status after PCI, whereas effects on death or myocardial infarction are neutral overall, underscoring the need for precise selection rather than a default interventional strategy.^3^,^6^ EuroCTO showed improved symptoms with PCI but no reduction in cardiovascular death or myocardial infarction versus optimal medical therapy on extended follow-up.^3^ DECISION-CTO, limited by crossover and early stopping, also showed no superiority of routine CTO-PCI for major adverse events, reinforcing the principle that precise patient selection should drive revascularisation strategy.^5^

Within that selection framework, myocardial viability is important because it predicts the likelihood of regional functional recovery, symptom relief and favourable remodelling once flow is restored.^7^ Cardiovascular magnetic resonance (CMR) is the reference non-invasive tool for viability assessment using late gadolinium enhancement (LGE) imaging. LGE-defined transmurality shows a clear inverse relation to post-revascularisation recovery.^8^ CMR can not only inform viability, but also help establish inducible ischaemia in the CTO territory precisely and is increasingly used to select patients for CTO-PCI.^8^,^10^ Translation into routine pathways, however, is variable because access and expertise differ, and because links between angiographic signals in CTO and tissue-level health remain incompletely defined.

Collateralisation is the key angiographic feature of chronic occlusion.^11^ Collaterals may maintain resting perfusion and limit scar, but in practice, we grade them with semiquantitative scales such as Rentrop that describe filling rather than true flow.^2 11 12^ Data linking collateral grade with viability are mixed: some CMR or radionuclide studies report that higher grades indicate more viable myocardium, whereas others show weak or no association with contemporary stress-CMR.^1013^,^15^ This uncertainty frames our question: can the Rentrop grade help predict CMR-defined viability in CTO and guide revascularisation decisions?

We hypothesised that higher Rentrop collateral grades would independently predict CMR-defined myocardial viability within the CTO territory and offer practical discriminatory performance for selecting candidates for revascularisation. The objectives were to quantify the association between Rentrop grade and viability; to test the independence of this relation after adjustment for clinical and angiographic covariates; and to evaluate diagnostic performance in identifying a collateral threshold that enriches for viability and can be operationalised in routine practice.

## Methods

### Study cohort

This retrospective observational cohort study included patients with a confirmed diagnosis of CTO who underwent CMR for myocardial viability assessment at Norfolk and Norwich University Hospitals NHS Foundation Trust between 2016 and 2021. A total of 56 patients fulfilled the study inclusion criteria.

Eligible participants were aged 18–90 years, had symptoms of stable or unstable angina and demonstrated at least one CTO in a major epicardial coronary artery (left anterior descending, left circumflex or right coronary artery) on invasive coronary angiography. All patients completed a stress perfusion CMR study. Patients were excluded if they had contraindications to CMR or gadolinium-based contrast agents, recent myocardial infarction (within 90 days), non-ischaemic cardiac pathology (including congenital or significant valvular disease) or advanced renal impairment.

CTO was defined as complete coronary occlusion with no antegrade flow (thrombolysis in myocardial infarction (TIMI) grade 0) of at least 3 months’ duration. Multivessel disease was considered present where ≥70% stenosis was identified in one or more non-CTO vessels. These definitions are consistent with those applied in our previous work on myocardial viability and ischaemia in CTO.^9^

### Coronary angiogram analysis

Coronary angiograms were reviewed independently and blinded to CMR findings by an experienced cardiology fellow (PS). The CTO vessel, TIMI flow grade, collateralisation (Rentrop score) and the presence of significant concomitant coronary artery disease were recorded.

### CMR acquisition protocol

All scans were conducted on a 1.5 T system (Siemens Healthineers, Erlangen, Germany). Cardiac synchronisation was achieved using prospective ECG gating with the manufacturer’s vector-ECG algorithm. Perfusion imaging was performed under free-breathing conditions, and all patients had intravenous access established for both adenosine administration and contrast delivery, in accordance with standard clinical protocol and have been described previously.^9^

The imaging protocol comprised cine acquisitions using balanced steady-state-free precession sequences (including 2-chamber, 3-chamber and 4-chamber views and a complete short-axis stack with 30 cardiac phases), followed by adenosine stress first-pass perfusion imaging and LGE for assessment of myocardial scar.

Pharmacological stress was induced with adenosine infused at 140 µg/kg⁻¹/min⁻¹ over 4 min. During peak hyperaemia, a single bolus of gadobutrol (1.0 mmol/mL⁻¹) was administered at a dose of 0.05 mmol/kg⁻¹ via an antecubital vein at 3 mL/s⁻¹, followed by a 20 mL saline flush. No pre-bolus technique or dual-sequence acquisition was used, allowing simultaneous capture of the arterial input function and myocardial enhancement within a single saturation-recovery gradient-echo acquisition. Imaging commenced after approximately 150 s of adenosine infusion once haemodynamic stability was achieved. Adequate stress was defined by either a rise in heart rate of ≥10 beats per minute, a reduction in systolic blood pressure of ≥10 mm Hg or the presence of typical adenosine-related symptoms.

First-pass perfusion imaging used a single-shot saturation-recovery gradient-echo (TurboFLASH) sequence with Generalized Autocalibrating Partially Parallel Acquisitions (GRAPPA) acceleration (R=2). Three short-axis slices (basal, mid-ventricular and apical) were obtained per cardiac cycle, with an in-plane resolution of 2.7×2.2 mm² and slice thickness of 8 mm. Sequence parameters included repetition time/echo time of 2.6/1.1 ms, a flip angle of 12° and a saturation-recovery time of 105 ms. The acquisition bandwidth was 651 Hz pixel⁻¹, and each slice had a temporal footprint of 188 ms. A total of 60 dynamic frames were acquired during the first-pass sequence.

LGE imaging was performed 6–10 min after contrast administration using a two-dimensional phase-sensitive inversion recovery gradient-echo sequence. Full left ventricular coverage was obtained with contiguous short-axis slices, supplemented by standard long-axis views (2-chamber, 3-chamber and 4-chamber), acquired during end-expiratory breath-holds with ECG gating. Typical imaging parameters included repetition time 2.93 ms, echo time 1.12 ms and flip angle 45°, with an acquired spatial resolution of 1.9×1.4 mm² (reconstructed to 1.4×1.4 mm²). Slice thickness was 8 mm without interslice gap, and the field of view ranged from 300 to 360 mm. Receiver bandwidth was set to 781 Hz pixel⁻¹. Prior to each LGE acquisition, a Look-Locker sequence was used to determine the optimal inversion time for nulling normal myocardium, typically between 260 and 320 ms at 1.5 T. Phase-sensitive reconstruction was applied to maintain contrast between blood and myocardium despite heart rate variability. Images were reconstructed inline and exported in Digital Imaging and Communications in Medicine (DICOM) format for subsequent offline quantitative scar analysis.

### CMR analysis and post-processing

All cine, LGE and first-pass perfusion images were analysed using the in-house MASS research software (Version 2022-EXP; Leiden University Medical Center, Leiden, the Netherlands). Post-processing was performed on contiguous short-axis stacks covering the entire left ventricle from base to apex throughout the cardiac cycle.

Left ventricular volumes and function were derived from cine images using semi-automated contouring of the endocardial and epicardial borders. Papillary muscles were excluded from the myocardial contours to avoid overestimation of myocardial mass and underestimation of scar extent. To ensure consistency between datasets, contour positioning was standardised using the right ventricular insertion points on basal short-axis slices as anatomical landmarks.

Scar assessment was performed on phase-sensitive inversion recovery LGE images. Fibrotic tissue was defined as regions with signal intensity greater than 5 SDs above that of a carefully selected area of remote, unaffected myocardium. This reference region was chosen to minimise contamination from artefact, partial volume effects or subtle enhancement.

Segmental analyses were conducted according to the American Heart Association 16-segment model, which was consistently applied across cine, LGE and perfusion datasets to enable direct regional comparison. Quantification of LGE was performed semi-automatically across the full short-axis stack, with total scar burden expressed in grams and assigned to corresponding myocardial segments. Within CTO-related territories, scar burden was further expressed as both absolute mass and percentage of segmental myocardial mass to allow more detailed regional assessment of fibrosis.

### Viability and ischaemia

Myocardial viability and ischaemia were assessed by integrating LGE and stress perfusion findings in accordance to current practice.^8 16^ A segment was defined as viable when LGE involvement was ≤50% of wall thickness, and non-viable when LGE exceeded 50%. Ischaemia was defined as stress-induced hypoperfusion in segments without LGE or with ≤50% transmural LGE. At the territory level, a region was considered viable if more than 50% of its constituent segments met the segmental viability criterion.

### Myocardial perfusion analysis

First-pass perfusion during adenosine-induced stress was visually assessed by consensus between two experienced readers (PG ZM). A perfusion defect was defined as an area exhibiting diminished contrast uptake during stress compared with rest. The burden of reversible ischaemia was determined by subtracting the mass of LGE-defined scar from the total stress perfusion defect mass.

Absolute quantitative perfusion analysis was performed using pixel-wise parametric mapping of the stress perfusion datasets. Myocardial blood flow (MBF, mL/min/g) was derived using a model-based deconvolution approach applied to the arterial input function and myocardial signal-intensity time curves. This methodology follows established consensus recommendations and standardised techniques for quantitative CMR perfusion analysis.^16 17^ Global and regional MBF measurements were obtained, including values within the coronary territory supplied by the CTO vessel.

### Statistical analysis

Statistical analyses were performed using MedCalc Statistical Software (V.20.215; MedCalc Software, Ostend, Belgium) and OriginPro (version 2023; OriginLab Corporation, Northampton, Massachusetts, USA). The distribution of continuous variables was assessed using the Shapiro-Wilk test. Continuous data are presented as mean±SD and compared among groups stratified by Rentrop collateral flow grade using one-way analysis of variance. Post hoc pairwise comparisons were conducted with the Tukey-Kramer test, and the Jonckheere-Terpstra trend test was applied to evaluate ordered associations of CTO territory, myocardial viability and perfusion parameters across Rentrop grades. Categorical variables are expressed as frequencies and percentages. The discriminative performance of the Rentrop score for predicting myocardial viability within CTO territories was evaluated using receiver operating characteristic (ROC) curve analysis. Stepwise logistic regression was used to identify independent predictors of myocardial viability within the CTO territory. All tests were two-tailed, and a p value<0.05 was considered statistically significant.

## Results

### Patient characteristics

The mean age of participants was comparable between patients without collateral flow and those with collateral flow (74.11±7.96 years vs 70.09±10.59 years; p=0.28), though this difference was not statistically significant. Males were more prevalent in the collateral group (66.7% vs 91.5%; p = 0.04) (table 1). In the total cohort, CTO was mainly observed in the right coronary artery, accounting for 57.1% of cases (32 out of 56). The left anterior descending artery was involved in 25% of patients (14 out of 56), while the left circumflex artery accounted for 17.8% (10 out of 56) (table 2).

**Table 1:** Baseline patient demographics and clinical characteristics

Coronary collateralisation	No collateral flow (n=9)	Collateral present with retrograde flow (n=47)	P value
Male (%)	6 (66.7)	43 (91.5)	0.04
Age (years)	74.11±7.96	70.09±10.59	0.28
Height (cm)	164.00±7.84	173.25±8.41	0.08
Weight (kg)	83.11±17.47	87.49±15.67	0.45
Body mass index (kg/m²)	29.24±6.12	29.22±5.33	0.99
Systolic blood pressure (mm Hg)	129.44±16.85	126.30±17.85	0.63
Diastolic blood pressure (mm Hg)	73.22±12.44	72.09±12.19	0.80
Haemoglobin (g/L)	125.22±30.00	135.66±19.77	0.19
Creatinine (µmol/L)	105.78±38.81	93.21±28.81	0.26
eGFR (mL/min/1.73 m²)	62.56±26.33	72.02±17.61	0.18
Lipid-lowering therapy (n, %)	4 (44.4)	25 (53.2)	0.63
Diabetes mellitus (n, %)	2 (22.2)	18 (38.3)	0.36
Hypertension (n, %)	4 (44.4)	23 (48.9)	0.81
Smoking history (n, %)	6 (66.7)	31 (66.0)	0.97
Atrial fibrillation (n, %)	3 (33.3)	5 (10.6	0.08
Cerebrovascular accident history (n, %)	1 (11.1)	3 (6.4)	0.62
Previous myocardial infarction (n, %)	7 (77.8)	36 (76.6)	0.94
Ventricular tachycardia (n, %)	1 (11.1)	6 (12.8)	0.89
CCS angina class	3.56±0.53	2.85±0.69	**0.01**
NYHA functional class	2.56±1.13	2.17±0.96	0.29
Left ventricular end-diastolic volume (mL)	194.62±54.17	196.33±51.88	0.93
Left ventricular end-systolic volume (mL)	107.99±43.72	105.44±43.92	0.87
Left ventricular stroke volume (mL)	86.62±20.44	90.90±20.81	0.57
Left ventricular ejection fraction (%)	46.36±10.67	47.69±10.36	0.73
Regional wall motion abnormality (%)	8 (89)	27 (57)	**0.02**
Left ventricular mass (g)	130.43±42.56	138.68±32.91	0.51
Right ventricular end-diastolic volume (mL)	132.70±42.83	158.63±32.16	**0.04**
Right ventricular end-systolic volume (mL)	49.04±25.10	67.86±18.29	**0.01**
Right ventricular stroke volume (mL)	83.66±23.01	90.77±25.01	0.43
Right ventricular ejection fraction (%)	65.00±11.00	56.94±8.79	**0.02**

All data are presented as mean±SD or n (%).

Comparison of variables between patients with and without collateral flow using independent t-test.

CCS, Canadian Cardiovascular Society; eGFR, estimated glomerular filtration rate; NYHA, New York Heart Association.

**Table 2:** Comparison of coronary territory characteristics, myocardial viability and perfusion parameters across Rentrop collateral grades

Rentrop score (grade)	Score 0 (n=9)	Score 1 (n=16)	Score 2 (n=27)	Score 3 (n=4)	P value*	P value†
LAD CTO (n, %)	4 (44.4)	5 (31.2)	4 (14.8)	1 (25.0)	0.27	0.05
LCx CTO (n, %)	3 (33.3)	2 (12.5)	5 (18.5)	0 (0.0)	–	–
RCA CTO (n, %)	2 (22.2)	9 (56.2)	18 (66.7)	3 (75.0)	–	–
Viability in CTO territory (n, %)	4 (44.4)	10 (62.5)	24 (88.9)	3 (75.0)	0.04	–
MBF in CTO territory (mL/min/g)	2.21±0.30	2.19±0.53	1.96±0.47	2.64±0.51	0.04	–
MBF in non-CTO territory (mL/min/g)	2.62±0.73	2.67±0.74	2.51±0.62	3.03±0.67	0.52	0.98
Scar tissue in CTO territory (%)	26.48±21.14	22.69±13.95	16.45±13.71	19.69±29.69	0.38	0.04

All data are presented as mean±SD or n (%).

Comparison of clinical, angiographic and imaging variables among groups stratified by Rentrop collateral flow grade.

* Post hoc Tukey-Kramer test was used for pairwise comparisons.

† Jonckheere-Terpstra trend test was applied to assess ordered associations of CTO territory, myocardial viability, perfusion parameters and myocardial scar across Rentrop grades.

CTO, chronic total occlusion; LAD, left anterior descending; LCx, left circumflex; MBF, myocardial blood flow; RCA, right coronary artery.

Baseline demographics including weight, body mass index, blood pressure, haemoglobin, creatinine and estimated glomerular filtration rate were comparable between groups (p>0.05 for all). Left ventricular characteristics, including end-diastolic and end-systolic volumes, stroke volume, ejection fraction and myocardial mass, were similar between groups without statistically significant differences. Although some variation was observed in right ventricular parameters, these differences may not be clinically relevant (table 1).

### Collateral coronary circulation (Rentrop scoring)

**Figure 1:**
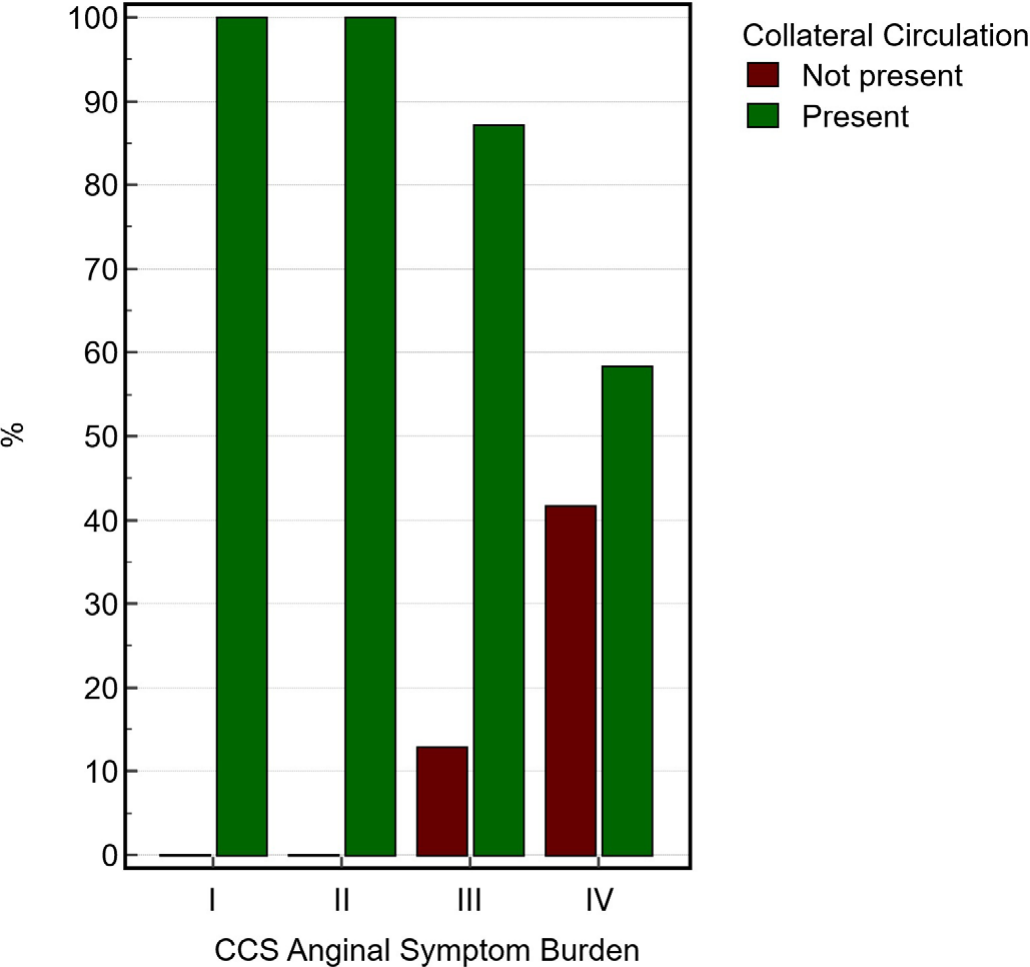
Histogram demonstrating how the presence of collateralisation in the CTO territory relates to patient CCS anginal symptom burden. Notably, all patients in classes I and II exhibited collateral circulation. Statistical analysis via χ² test demonstrated a moderate association between CCS class and collateral status (χ²=8.5, df=3, p=0.03). CCS, Canadian Cardiovascular Society; CTO, chronic total occlusion.

Patients classified in CCS I and II demonstrated 100% prevalence of collateral circulation, with no individuals lacking collaterals. In CCS III, 87.0% exhibited collaterals while 13.0% did not. In CCS IV, collateral circulation was observed in 58.5% of individuals, with 41.5% lacking collaterals (table 1). The χ^2^ analysis showed a statistically significant association between CCS anginal burden and collateral presence (χ²=8.55, df=3, p=0.04), yielding a contingency coefficient of 0.36, denoting a moderate correlation (figure 1).

**Figure 2:**
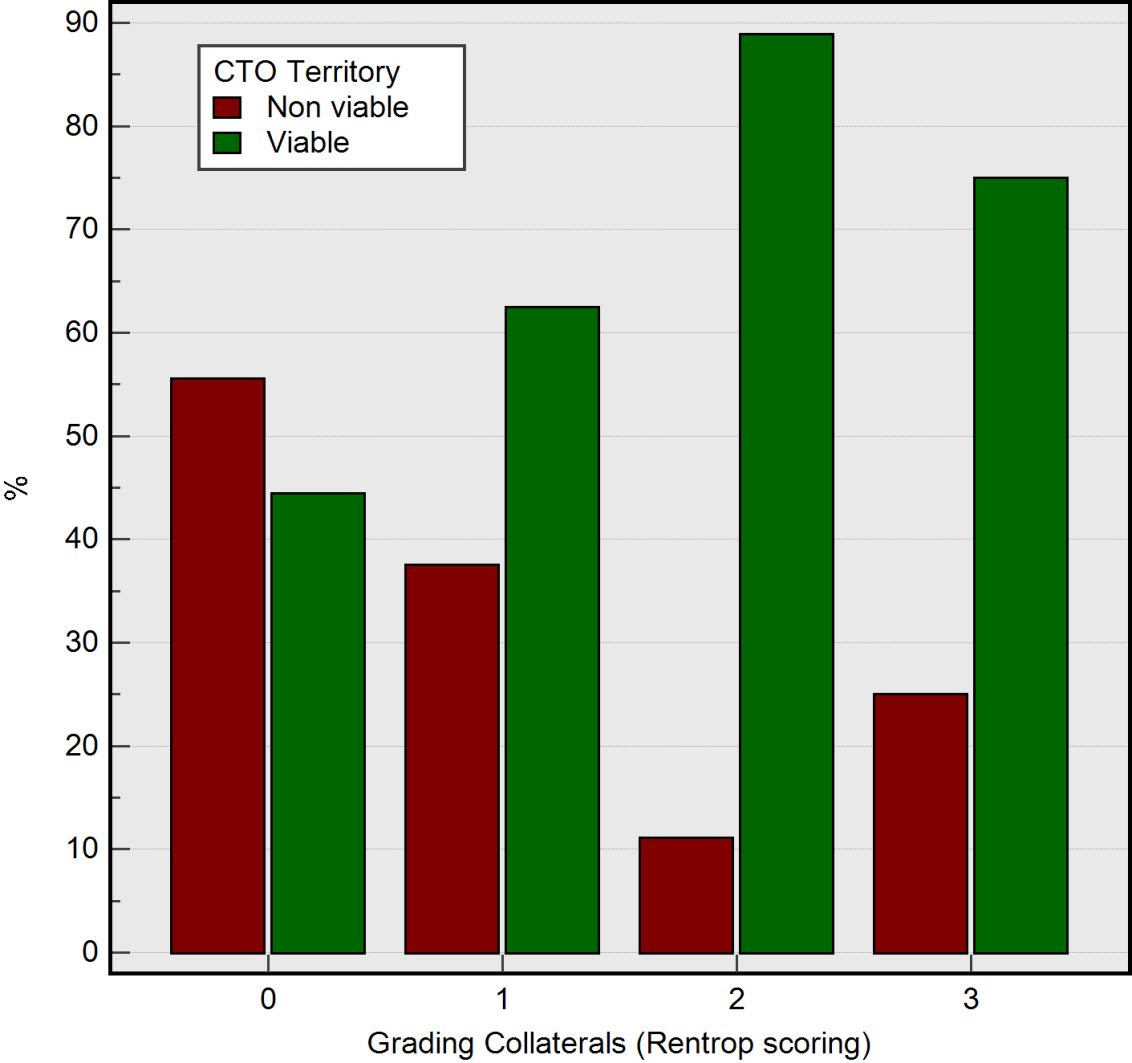
Histogram demonstrating how Rentrop-graded collateralisation relates to myocardial viability within the CTO territory. CTO, chronic total occlusion.

Among the study groups stratified by Rentrop score for coronary collateral flow, the presence of significant myocardial viability in chronic total occlusion territory was more frequent in higher Rentrop scores (p=0.04) (table 2). Among patients with Rentrop score 0, 55.6% had non-viable myocardium while 44.4% were viable. For Rentrop score 1, non-viable and viable territories were observed in 37.5% and 62.5%, respectively. A further increase in collateral grade (Rentrop score 2) showed a marked predominance of viable myocardium (88.9%), with only 11.1% being non-viable. At the highest collateral grade (score 3), 75.0% of cases exhibited viability, with 25.0% being non-viable. The χ^2^ test indicated a statistically significant association between Rentrop score and myocardial viability (χ²=8.12, df=3, p=0.04), with a contingency coefficient of 0.36, denoting a moderate effect size (figure 2).

Myocardial blood flow in chronic total occlusion territory demonstrated a statistically significant difference across Rentrop groups (p=0.04), while no significant differences were observed in myocardial blood flow in non-chronic total occlusion territory (p=0.52; trend p=0.98). The percentage of scar tissue in chronic total occlusion territory demonstrated a trend with high Rentrop score resulting in lesser scar (p=0.04) (table 2).

In a stepwise logistic regression analysis, Rentrop grading of coronary collateral flow was identified as the sole significant predictor of myocardial viability in the chronic total occlusion territory (p=0.012), with each incremental increase in Rentrop score associated with more than a twofold increase in the odds of viability (OR 2.52, 95% CI 1.18 to 5.42). The overall model demonstrated moderate discriminative performance, with a classification accuracy of 75.0% and an area under the receiver operating characteristic curve of 0.71 (95% CI 0.57 to 0.82). Model fit was acceptable as per the Hosmer-Lemeshow test (p=0.27). Notably, angiographic flow (TIMI grade) and symptom severity at the time of chronic total occlusion assessment (Canadian Cardiovascular Society angina class) were not retained in the final model (table 3).

**Table 3:** Logistic stepwise regression summary: predicting myocardial viability in CTO (n=56)

Variable category	Variable	Included in model	Coefficient	P value	OR (95% CI)
Symptom severity	CCS functional class (prior to CTO evaluation)	✘	–	–	–
Angiographic findings	TIMI flow grade in CTO territory	✘	–	–	–
Collateral circulation	Rentrop grading of coronary collateral flow	✔	0.93	0.018	2.52 (1.18 to 5.42)
**Model performance:** χ²(1)=6.26, p=0.012, Nagelkerke R²=0.15, classification accuracy=75.0%, AUC=0.71 (95% CI 0.57 to 0.82), Hosmer-Lemeshow p=0.27.					

AUC, area under the curve; CCS, Canadian Cardiovascular Society; CTO, chronic total occlusion; TIMI, thrombolysis in myocardial infarction.

**Figure 3:**
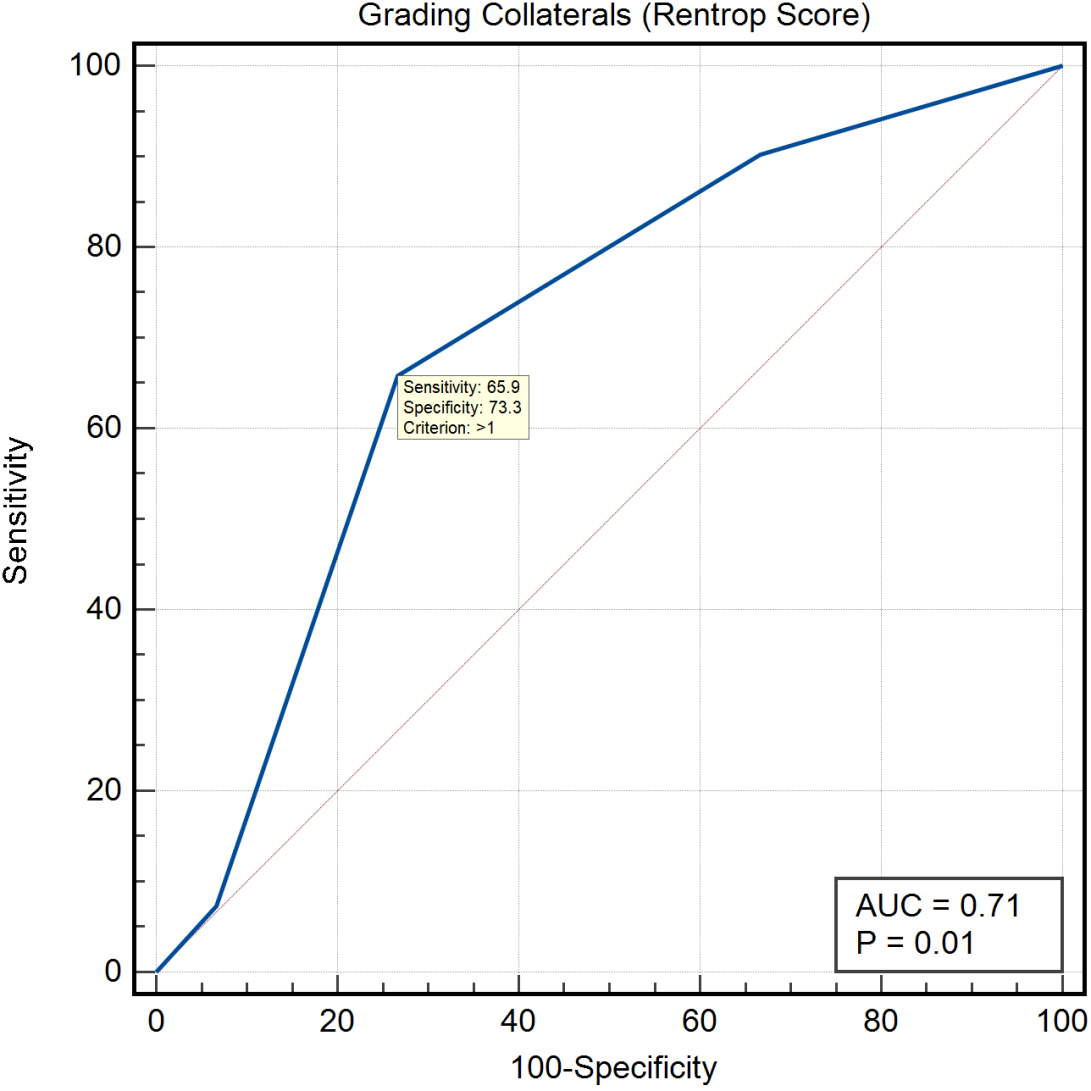
ROC curve depicting the diagnostic performance of Rentrop-based grading of collateral circulation for predicting myocardial viability within chronic total occlusion territories. The curve demonstrates an area under the ROC curve (AUC ROC) of 0.71 with a p value of 0.01, indicating a statistically significant ability to distinguish between viable and non-viable myocardial segments. AUC, area under the curve; ROC, receiver operating characteristic.

ROC curve analysis for diagnostic performance of the Rentrop score revealed an area under the curve (AUC ROC) of 0.71, with an SE of 0.08 and a 95% CI ranging from 0.57 to 0.82. The z statistic was 2.5 (p=0.01). The optimal diagnostic threshold, as defined by the maximum Youden index, was identified at a Rentrop score greater than one. At this cut-off, the Youden index was 0.39, sensitivity was 65.85% and specificity was 73.33% (figure 3).

## Discussion

**Figure 4:**
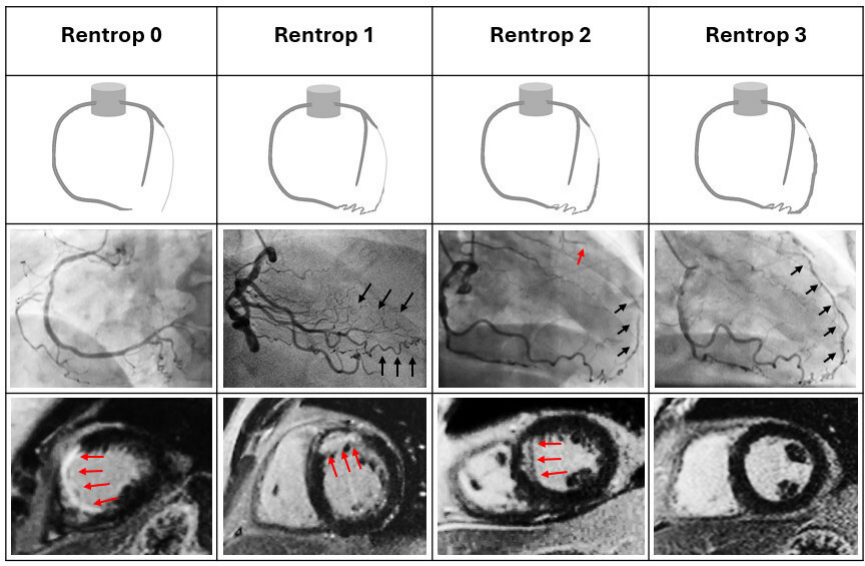
Schematic and coronary angiographic examples illustrating collateral vessel grading using the Rentrop classification, alongside short-axis LGE images of the left ventricle demonstrating the presence of myocardial scar. Rentrop 0: LAO projection demonstrating no collateral flow from the right coronary artery. CMR LGE demonstrating transmural scar of the septum and anterior LV segments (red arrows). Rentrop 1: RAO view of RCA with collaterals supplying the LAD branches (black arrows), with the occluded epicardial LAD segment not visualised or filled. LGE shows scar involving 70–80% the myocardial wall thickness in the anterior LV wall (red arrows). Rentrop 2: PA cranial view of RCA depicting partial epicardial filling of the occluded LAD (black arrow). CTO site in proximal LAD marked with the red arrow. LGE demonstrates subendocardial scar in antero-septum involving <50% of wall thickness (red arrows). Rentrop 3: RAO view of the RCA shows full collateral perfusion of the occluded LAD (black arrows). CMR demonstrates no myocardial scar on late gadolinium enhancement. CMR, cardiovascular magnetic resonance; CTO, chronic total occlusion; LAD, left anterior descending; LAO, left anterior descending; LGE, late gadolinium enhancement; LV, left ventricular; PA, Posteroanterior; RCA, right coronary artery; RAO, right anterior oblique.

We set out to test whether a simple angiographic signal—the Rentrop collateral grade—could estimate the likelihood of CMR-defined myocardial viability within the CTO territory and support real-time decisions in the catheter laboratory. We observed a clear, graded relation: higher Rentrop grades aligned with progressively greater viability, whereas non-viable myocardium clustered at grades 0–1. A practical threshold emerged at Rentrop >1, identifying territories more likely to be viable and providing a straightforward rule to integrate with symptoms and ischaemia when considering CTO-PCI (figure 4). Collateralisation also tracked clinical status, being more frequent with milder angina and less frequent with severe symptoms, underlining its clinical relevance. Together, these findings show that the diagnostic angiogram offers decision-ready information: collateral grade turns anatomy into a probability of benefit, guiding when to proceed, when to request CMR and when to defer.

In contrast to our finding of a graded increase in CMR-defined viability with higher Rentrop scores and moderate diagnostic discrimination, the multicentre stress-CMR study by Pica *et al* reported no association between collateral presence/extent and either viability or ischaemia.^15^ Several methodological features likely account for this divergence. First, collateralisation was dichotomised as ‘well-developed’ (Rentrop 3 or Werner collateral connection grading of 2) versus ‘poorly developed’ (Rentrop 0/1 or Werner collateral connection grading of 0/1), and the primary analyses were driven by this binary split rather than an ordinal Rentrop model; such pooling of Rentrop and CC scales reduces granularity and can mask a dose-response signal. Second, viability definitions varied across subgroups: patients assessed with adenosine were deemed viable if the mean transmural LGE in the CTO territory was ≤50%, whereas those undergoing dobutamine relied on low-dose dobutamine contractile reserve irrespective of LGE. Mixing territory-mean scar thresholds with segmental contractile reserve introduces non-uniform misclassification and may dilute links with angiographic grades. Third, the registry treated other non-CTO lesions before CMR and mandated a 3-month optimisation period, potentially altering perfusion, symptoms and collateral recruitment before viability testing. Finally, the cohort had preserved global function (mean left ventricular ejection fraction (LVEF) 57%) and a high baseline prevalence of viability (≤50% LGE in 61%), compressing the dynamic range to detect collateral-related differences. Together, these design choices offer plausible reasons why a Rentrop viability relation was not observed in that study, whereas our uniform CMR viability definition and ordinal collateral analysis revealed a consistent graded pattern that is biologically coherent with collateral-mediated myocardial preservation.

A few studies align with our observations. Similar to Choi *et al*, who recruited 170 patients with CTO, we found that better collateralisation was associated with a more favourable tissue profile. In our data, this translated into a stepwise rise in CMR-defined viability as Rentrop grade increased, whereas Choi *et al* centred on scar burden and wall motion and treated collaterals as a binary exposure, limiting assessment of a dose-response relation.^13^ Also, their LGE quantification method differed from ours (LGE method: ours Phase Sensitive Inversion Recovery (PSIR) with 5 SD threshold; Choi *et al* 6 SD threshold).^13^ Schumacher *et al*^14^ reported less scar and greater wall thickening with well-developed collaterals, but defined ‘viability’ only in dysfunctional segments with ≤50% LGE and dichotomised collateral status by a combined CC2 + Rentrop 3 rule; this coupling of viability to dysfunction and loss of ordinal information likely explains their paradoxical excess of ‘viable’ myocardium in poorly developed collaterals and the absence of a positive Rentrop–viability gradient. Also, Schumacher *et al* provided percentage-transmurality categories in QMass without an explicit intensity threshold, which limits direct translation. Chen *et al*, in a prospective cohort of 47 patients, focused on peri-infarct border zone and strain metrics rather than viability and dichotomised collaterals at Rentrop 3; the small, high-LVEF sample and border-zone endpoint limit direct comparability to a territory-level viability outcome.^10^ Taken together, prior studies support links between coronary collateralisation and viability but are methodologically heterogeneous.

### Clinical implications

In the catheter laboratory, often the angiogram is the only imaging available. Using the full Rentrop scale informs downstream viability in the CTO territory: a Rentrop score >1 indicates a higher likelihood of CMR-defined viability. This offers a practical table-side rule: when symptoms and ischaemia are concordant and Rentrop >1, proceeding with CTO-PCI is reasonable; when Rentrop ≤1 and clinical benefit is uncertain, defer, optimise medical therapy and consider organising CMR. Because our viability endpoint is territory-level and independent of resting dysfunction, the collateral signal is not masked by preserved wall motion. The threshold sharpens consent and case selection, helps avoid long, low-yield procedures and directs CMR to cases where it will change management.

### Limitations

Several limitations of our study merit consideration. The retrospective and single-centre design introduces potential selection bias and limits the generalisability of our findings to broader populations. The sample size, though sufficient for detecting moderate associations, remains relatively small, particularly within subgroups stratified by Rentrop grade, which may reduce statistical power and the precision of estimates.

Rentrop grading, while widely used, is a semi-quantitative, two-dimensional assessment of collateral flow that does not capture microvascular perfusion, collateral geometry or dynamic haemodynamic parameters. Interobserver variability, although mitigated by blinded independent review, remains inherent to visual angiographic scoring and may have influenced results. Furthermore, CMR was performed at a single time point, precluding evaluation of temporal changes in myocardial viability, perfusion or remodelling, as might occur following CTO revascularisation. Consequently, the study cannot address whether baseline collateral grade predicts functional recovery after intervention. Similarly, pharmacological stress perfusion provides only a snapshot of myocardial blood flow under hyperaemia, and quantitative perfusion may be influenced by heart rate, blood pressure or loading conditions at the time of acquisition.

Although we excluded patients with acute myocardial infarction within 90 days and non-ischaemic cardiomyopathy, unmeasured comorbidities or prior silent infarcts could have affected regional myocardial viability, introducing potential confounding. Additionally, the use of a 1.5 T CMR system with standard resolution sequences, while consistent, may be less sensitive for detecting subtle subendocardial scar or microvascular dysfunction compared with higher-field strength scanners or emerging quantitative mapping techniques. Finally, our findings reflect associations rather than causality. While Rentrop grade independently predicted myocardial viability in multivariable analysis, the moderate discriminative performance (AUC ≈ 0.71) indicates that angiographic assessment alone cannot fully substitute for comprehensive tissue characterisation by CMR. Prospective studies with larger, multicentre cohorts, serial imaging and integration of advanced quantitative angiographic or haemodynamic measures are needed to validate and extend these observations.

## Conclusion

Angiographic grading of collaterals using the Rentrop score independently predicts myocardial viability in patients with CTO. These findings support integrating collateral assessment into risk stratification and revascularisation planning, particularly when advanced imaging is limited.

## Data Availability

Data are available upon reasonable request.
